# Tracing the spatiotemporal phylodynamics of Japanese encephalitis virus genotype I throughout Asia and the western Pacific

**DOI:** 10.1371/journal.pntd.0011192

**Published:** 2023-04-13

**Authors:** Fan Li, Yun Feng, Guowei Wang, Weijia Zhang, Shihong Fu, Zuosu Wang, Qikai Yin, Kai Nie, Juying Yan, Xuan Deng, Ying He, Liang Liang, Songtao Xu, Zhenhai Wang, Guodong Liang, Huanyu Wang

**Affiliations:** 1 Department of Arboviruses, NHC Key Laboratory of Biosafety, National Institute for Viral Disease Control and Prevention, State Key Laboratory for Infectious Disease Prevention and Control, Chinese Center for Disease Control and Prevention, Beijing, PR China; 2 Yunnan Provincial Key Laboratory for Zoonosis Control and Prevention, Yunnan Institute of Endemic Disease Control and Prevention, Dali, PR China; 3 Ningxia Medical University, Yinchuan, PR China; 4 Liaoning Center for Disease Control and Prevention, Shenyang, PR China; 5 Zhejiang Center for Disease Control and Prevention, Hangzhou, PR China; 6 Guangxi Zhuang Autonomous Region Center for Disease Control and Prevention, Nanning, PR China; 7 Department of Neurology, General Hospital of Ningxia Medical University, Engineering Research Center for Diagnosis and Treatment of Ningxia Nervous System Diseases, Yinchuan, PR China; University of Pittsburgh, UNITED STATES

## Abstract

**Background:**

Japanese encephalitis virus (JEV; *Flaviridae*: *Flavivirus*) causes Japanese encephalitis (JE), which is the most important arboviral disease in Asia and the western Pacific. Among the five JEV genotypes (GI–V), GI has dominated traditional epidemic regions in the past 20 years. We investigated the transmission dynamics of JEV GI through genetic analyses.

**Methods:**

We generated 18 JEV GI near full length sequences by using multiple sequencing approaches from mosquitoes collected in natural settings or from viral isolates obtained through cell culture. We performed phylogenetic and molecular clock analyses to reconstruct the evolutionary history by integrating our data with 113 publicly available JEV GI sequences.

**Results:**

We identified two subtypes of JEV GI (GIa and GIb), with a rate of 5.94 × 10^−4^ substitutions per site per year (s/s/y). At present, GIa still circulates within a limited region, exhibited no significant growth, the newest strain was discovered in China (Yunnan) in 2017, whereas most JEV strains circulating belong to the GIb clade. During the past 30 years, two large GIb clades have triggered epidemics in eastern Asia: one epidemic occurred in 1992 [95% highest posterior density (HPD) = 1989–1995] and the causative strain circulates mainly in southern China (Yunnan, Shanghai, Guangdong, and Taiwan) (Clade 1); the other epidemic occurred in 1997 (95% HPD = 1994–1999) and the causative strain has increased in circulation in northern and southern China during the past 5 years (Clade 2). An emerging variant of Clade 2 contains two new amino acid markers (NS2a-151V, NS4b-20K) that emerged around 2005; this variant has demonstrated exponential growth in northern China.

**Conclusion:**

JEV GI stain circulating in Asia have shifted during the past 30 years, spatiotemporal differences were observed among JEV GI subclade. GIa is still circulating within a limited range, exhibite no significant growth. Two large GIb clades have triggered epidemics in eastern Asia, all JEV sequences identified in northern China during the past 5 years were of the new emerging variant of G1b-clade 2.

## Author summary

Although Japanese encephalitis virus (JEV) genotype I (GI) has circulated in Asia and the western Pacific during the past 30 years, little is known regarding its evolution, geographical distribution, and epidemiology, especially the recent years. To address this knowledge gap, we performed a phylogenetic analysis using all full open reading frame JEV sequences assembled thus far, including 113 viral sequences sampled from eight countries during the past 42 years. We revealed spatiotemporal differences in spread among JEV GI subclades; identified amino acid markers for different clades; made an in-depth study on the evolution of GIb, described the geographical distribution of the recent active GIb clade 2 and identified its emerging variant.

## Introduction

Japanese encephalitis (JE) is caused by a mosquito-borne flavivirus, Japanese encephalitis virus (JEV), which is the most common etiology of viral encephalitis in Asia and the western Pacific (particularly in China) [1, 2]. JE was first documented in 1871 in Japan [3]. An estimated 68,000 clinical cases of JE are recorded globally each year, with approximately 13,600–20,400 deaths. JE primarily affects children [1]. Most adults in endemic countries have natural immunity after childhood infection; however, individuals of any age can be affected [1]. There is a risk of JEV transmission in 24 countries of Southeast Asia and the western Pacific, a region that includes more than 3 billion people [1, 3]. JEV is transmitted to humans through bites from infected mosquitoes of the *Culex* genus (mainly *Culex tritaeniorhynchus*). After infection, humans do not develop sufficient viremia to infect feeding mosquitoes. The enzootic transmission cycle of the virus typically involves mosquitoes, pigs, and/or water birds [3].

JEV has a single-stranded RNA genome that contains only an open reading frame (ORF) flanked by the 5’ and 3’ untranslated regions [4]. The ORF encodes three structural proteins [capsid (C), pre-membrane (prM), and envelope (E) proteins] and seven non-structural (NS) proteins (NS1, NS2a, NS2b, NS3, NS4a, NS4b, and NS5) [5]. JEV is classified into five distinct genotypes (GI–V) based on phylogenetic analysis of the viral envelope gene or the complete genome [6, 7]. JEV was first isolated in Japan in 1935, this strain was later identified as GIII [8, 9], which was the most common genotype in regions where JE was endemic prior to 2000 [10]. The earliest JEV GI strain was collected from mosquito in Cambodia in 1967 [9], GI have been increasingly isolated from *Culex tritaeniorhynchus*, stillborn piglets, and JE patients in 1990s; thus, JEV GI has replaced JEV GIII as the dominant genotype in Japan, Korea, Vietnam, Thailand, China, and India during the past 20 years [11–15]. JEV GII and GIV strains are mainly found in Malaysia and Indonesia, although they do not cause mass epidemics [9]. The first known JEV GV strain, the Muar strain, was isolated from brain tissue specimens from viral encephalitis patients in Malaya in 1952 [16]. Since then, JEV GV strains have been re-isolated from mosquitoes in China and South Korea; they have been isolated from humans in South Korea [17–20].

Although GI is the dominant JEV genotype in traditional epidemic regions of Asia, recent years have seen changes in epidemiological and clinical features of JE in northern China, many aspects of JEV’s history, biology, and ecology remain poorly understood [21–24]. JEV evolution has been previously studied using phylogenetic methods [6, 7, 12, 25–28]; however, there has been no in-depth analysis of the molecular evolution of the latest JEV GI stains that incorporates the complete genetic diversity of the virus (represented by full length genomes). Here, we performed a phylogenetic analysis of JEV GI using genomes obtained from GenBank, by integrating newly generated GI sequences recently sampled in China.

## Materials and Methods

### Sampling and sequencing of JEV isolates from China

Mosquitoes collection and virus isolation protocols have been described elsewhere [21]. RNA was extracted using the QIAamp viral RNA mini kit (Qiagen, Hilden, Germany) and reverse-transcribed using SuperScript III reverse transcriptase (Invitrogen, Waltham, MA, USA), in accordance with the manufacturer’s instructions. JEV genomes were sequenced using samples from mosquitoes homogenized or JEV isolated through cell culture by Sanger method and Next generation sequencing (NGS). cDNA sequencing libraries were prepared using a Nextera XT kit (Illumina, San Diego, CA, USA), and 150 bp of paired-end sequencing was performed using a MiniSeq system (Illumina). The raw paired-end reads obtained from high-throughput sequencing were subjected to quality control using the Trimmomatic (v0.39), BBDUK (v38.79), and Fastp (v0.20.0) tools. Adapters, low-complexity reads, and low-quality bases (with scores less than 20) were removed to generate clean data. Then, the clean reads were mapped to reference sequences (GenBank accession number: HM366552) using the BWA-MEM aligner [29, 30]. Final consensus sequences were generated using iVar software [31]. Gaps were filled by PCR and Sanger sequencing. The new sequences were deposited in GenBank under accession nos. OM572533–OM572550 (S1 Table).

### Data

In addition to the newly generated full-genome sequences, we collected all available full length sequences of the JEV GI genome from GenBank at the National Center for Biotechnology Information. We excluded all sequences that lacked a sampling location, date and host. Then we analyzed potential recombination events using RDP, GENECONV, Chimaera, MaxChi and Bootscan methods implemented in the RDP Beta 5.27 software; Potential recombination events were identified by at least four methods and manually confirmed, then removed from further analysis. Hyper-mutations and low-quality sequences were also removed. The GenBank accession numbers of all sequences used in this study are listed in S1 and S2 Tables. These GenBank sequences represent the genetic diversity of JEV GI circulating in Asia; they cover the complete sampling history of the virus from the mid-1970s to 2019.

Prior to sequence alignment, the untranslated regions of each segment were removed and the ORF was identified. Sequences were aligned using the MUSCLE algorithm [32]. The alignment was manually checked and edited to ensure codon alignment.

### Molecular phylogenetics and molecular clock analyses

To explore the rates of JEV molecular evolution and evaluate the temporal signal, we compared tip-to-root genetic distances in the maximum likelihood tree against the sampling dates of the corresponding sequences, using the approach implemented in TempEst v1.5.3 software (S1 Fig). Regression plots were visually inspected to identify notable outliers, which were removed prior to further analysis. The linear regression correlation coefficient was used as a measure of the degree to which sequences evolved in a clock-like manner.

We estimated the evolutionary history of JEV GI by constructing time-calibrated maximum clade credibility (MCC) trees for the full ORF. To estimate time-calibrated phylogenies dated from time-stamped genome data, we conducted phylogenetic analysis using a Bayesian software package [33]. Substitution model selection was performed by IQ-TREE [34]. Other models were set as recommended by previous research [26]. We used the general time-reversible substitution model; codon position partitioning was performed using a gamma distribution model of among-site heterogeneity and the Bayesian skyline tree prior [35], with an uncorrelated relaxed clock that exhibited log-normal distribution [36]. To avoid potential sampling bias effects on ancestral reconstruction, we performed discrete trait ancestral reconstruction [37]. The analyses were conducted in duplicate using BEAST v1.10.4 software for 200 million Markov chain Monte Carlo steps, sampling parameters, and trees at every 10,000th step. All parameters have the effective sample size (ESS) values larger than 200. Convergence of the Markov chain Monte Carlo chains (MCMC) was checked using Tracer v1.7.3 software. MCC trees were summarized using TreeAnnotator software after discarding 10% as burn-in. Spatiotemporal evolutionary analysis based on the MCC trees with discrete trait was performed using Spread D3 software.

### Selection analyses

The C, prM, E, NS1, NS2a, NS2b, NS3, NS4a, NS4b, and NS5 ORF alignments were tested for evidence of natural selection using various methods available in HYPHY software [38], implemented on the Datamonkey 2.0 server [39]. Statistical tests (*p<0*.*05*) were conducted to evaluate evidence for pervasive and episodic selection in the JEV genome; they also were used to evaluate selection across whole genes and at specific codons. Analyses of pervasive selection were performed using three methods: Branch site Unrestricted Statistical Test for Episodic Diversification (BUSTED), which tests for gene-wise selection and estimates a mean dN/dS ratio for each gene; Single-Likelihood Ancestor Counting (SLAC); and Fixed-Effects Likelihood (FEL). The SLAC and FEL approaches were used to infer specific codons under adaptive selection. Episodic selection was evaluated using two methods: Mixed Effects Model of Evolution (MEME), which identifies individual codons under positive selection; and adaptive Branch-Site Random Effects Likelihood (aBSREL), which was used to search for phylogenetic branches under selection. A final analysis was performed using the Fast Unconstrained Bayesian Approximation (FUBAR) method to evaluate the difference between non-synonymous (β) and synonymous (α) rates per site for each viral segment. All codon positions were numbered relative to the JEV reference genome (NC_001437).

## Results

### Phylodynamics of JEV GI epidemics

**Fig 1:**
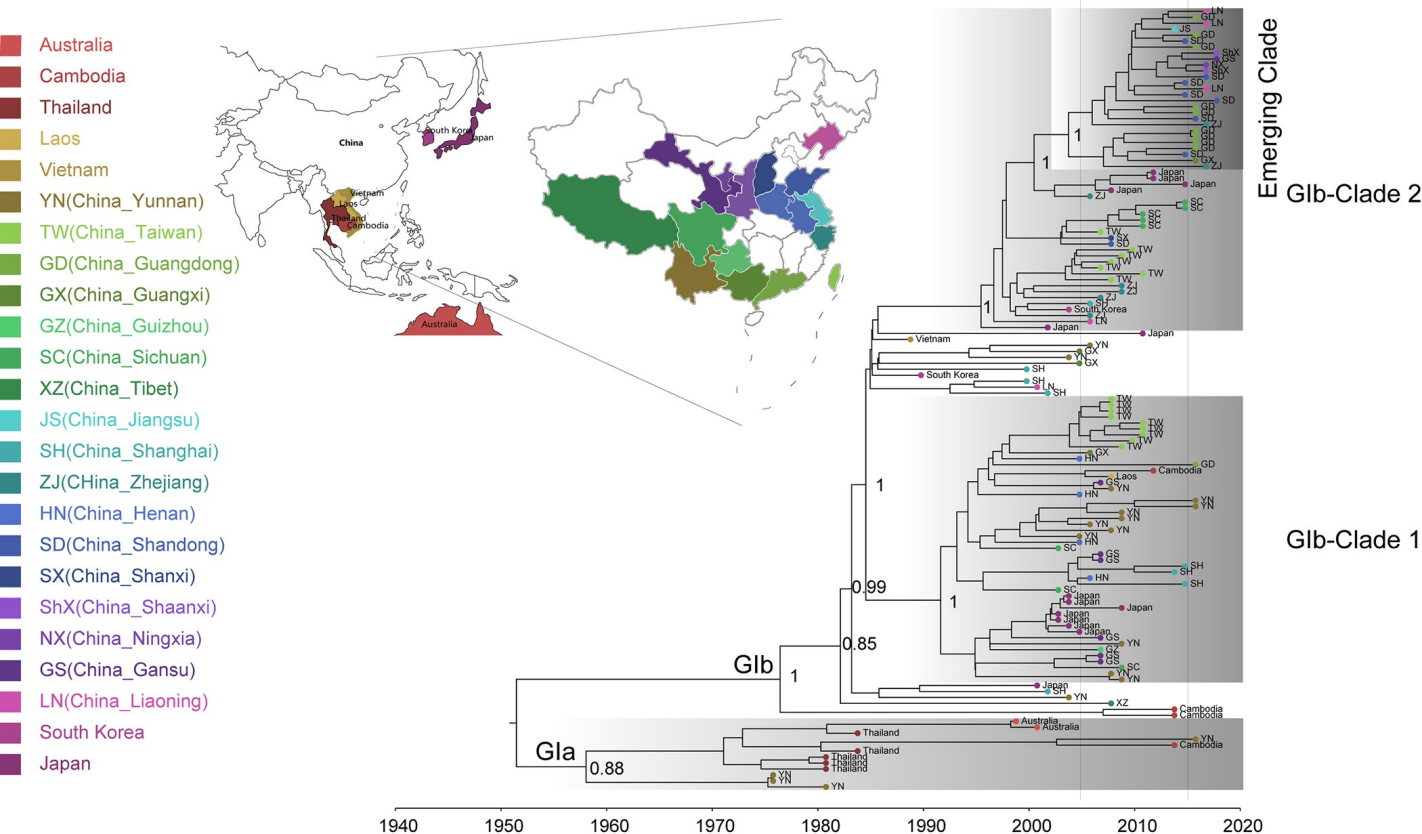
Time-scaled phylogeographic history of epidemic Japanese encephalitis virus (JEV) genotype I (GI). Boxes with gradient shading indicate clades GIa and GIb; posterior probabilities for their ancestral nodes are shown. Tip colors represent different sampling locations, according to the map at upper left. The base layer of the modified maps are sourced from Natural Earth, and download in GeoJSON format from website (https://geojson-maps.ash.ms/).

**Fig 2:**
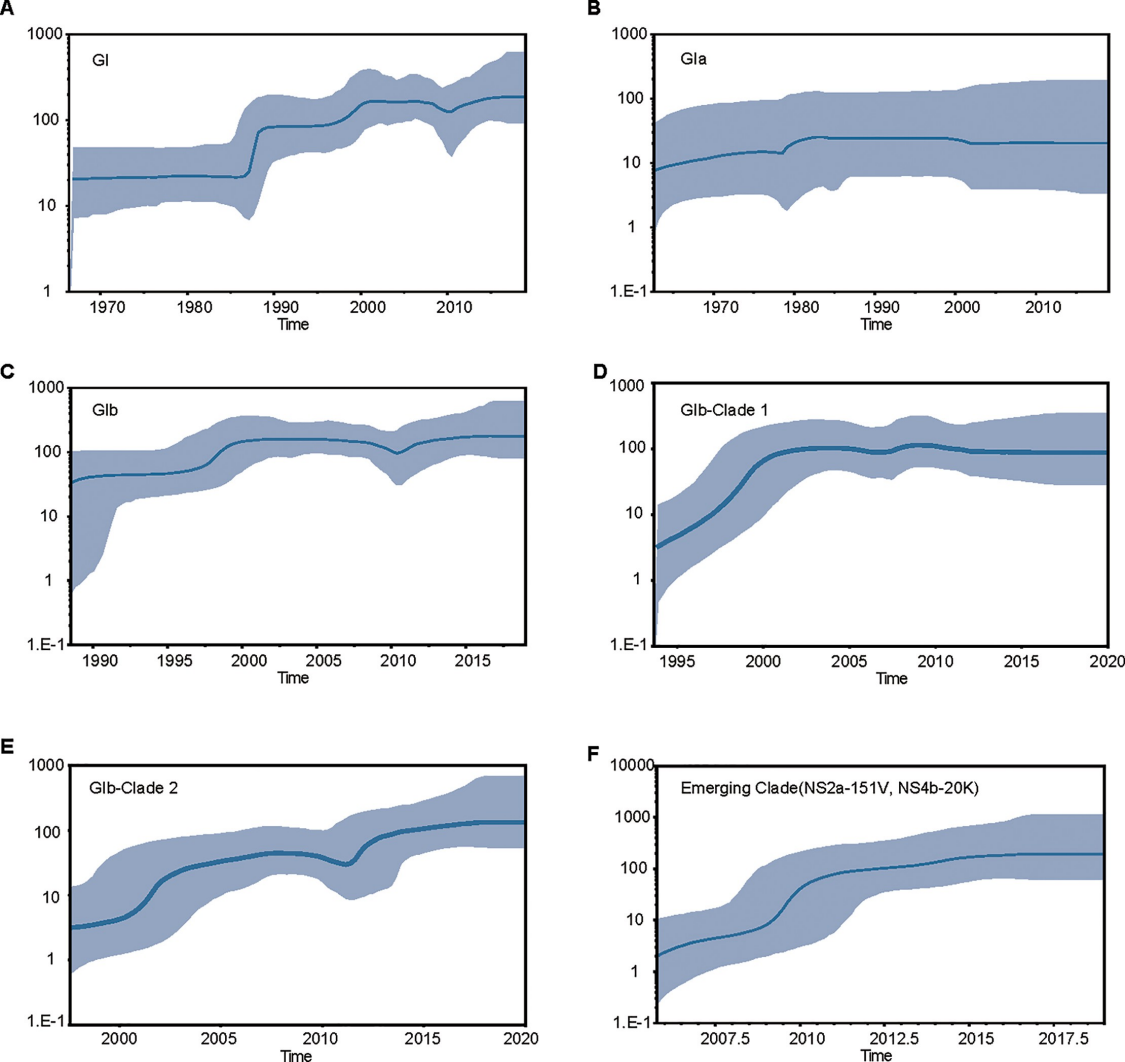
Bayesian skyline plots showing the demographic history of JEV GI including (A) GI, (B) GIa, (C) GIb, (D) GIb clade 1, (E) GIb clade 2, and (F) sub-clades NS2a-151V and NS4b-20K. Each y-axis represents virus effective population size (Ne), which is a measure of genetic diversity based on the number of genomes effectively contributing to new infections. Each x-axis represents time in calendar years. Blue lines are median estimates; purple lines are 95% highest posterior density (HPD) intervals.

The final alignment included 113 published sequences plus 18 new sequences. We analyzed the dataset containing 131 JEV sequences from 1977 to 2019, which covered eight countries (Thailand, Cambodia, South Korea, Vietnam, Japan, Laos, Australia, and China) and 17 Chinese provinces or regions of South East Asia and the western Pacific (Liaoning, Shanxi, Shandong, Shaanxi, Gansu, Ningxia, Henan, Jiangsu, Zhejiang, Shanghai, Tibet, Sichuan, Guizhou, Yunnan, Guangxi, Guangdong, and Taiwan). To investigate the spatial origins of JEV GI, we jointly considered the phylogenetic and ancestral location uncertainties of the discrete phylogeographic framework, which consistently identified regions near Thailand [location posterior probability = 0.41] or Yunnan Province in China (posterior probability = 0.27) as the ancestral locations of JEV genotype GI diversity (Figs 1 and S1; S3 Table). We inferred a median evolutionary rate of 5.94 × 10^−4^ [95% Bayesian credible interval (BCI): 5.03–6.94 × 10^−4^] s/s/y, using a coalescent model. This result is similar to previously reported estimates [25, 40]. We estimated the time of the most recent common ancestor (TMRCA) of GI to be around 1951 (95% BCI: 1937–1966; Figs 1 and S2). A Bayesian skyline plot of the effective population size of the total JEV GI dataset is shown in Fig 2; it indicates constant JEV GI growth. The JEV GI epidemic remained in a steady non-expansion phase from 1950 to 1980, followed by two exponential phase in the late 1980s and 1990s, constant growth after 2000, and a steady non-expansion phase (Fig 2A).

### Identification of independent JEV GI clades

Consistent with previous reports [25], we identified the GIa and GIb subtypes based on the MCC tree (Fig 1). The initial GIa derived from this common ancestor circulated within Thailand or Yunnan Province in China (Fig 1). GIa did not cause mass epidemics; it appears to have remained geographically limited to Yunnan Province, Thailand, Australia, and Cambodia within a restricted local area. The Bayesian skyline plot of the effective population size of the JEV GIa dataset is shown in Fig 2. JEV GIa groups exhibited no significant growth during the past 40 years (Fig 2B); the clade spread southward and then emerged in Australia in 2002 and Cambodia in 2015. GIa was re-isolated in Yunnan Province in 2017.

In contrast, the second phase of JEV GI spread was characterized by long-distance movement events and epidemics of the GIb strain in most regions of East Asia, particularly China (Fig 1). Our analyses strongly supported Cambodia as the spatial origin of the JEV GIb epidemic (posterior probability = 0.69; Figs 1 and 2C). The genetic histories of the JEV full ORF revealed two large GIb clades responsible for regional epidemics in China (Fig 1). A Bayesian skyline plot of the effective population size of the JEV GIb dataset is shown in Fig 2. Exponential growth of JEV GIb was observed when its inception in the mid-1980s and late 1990s (Fig 2C).

GIb Clade 1 originated in 1992 (95% HPD: 1989–1995) and circulated mainly in southern China (Yunnan, Shanghai, Guangdong, and Taiwan), where it has remained for the past decade with the activity decreased. Clade 2 underwent a larger expansion and is now distributed across northern and southern China, with a TMRCA of 1997 (95% HPD: 1994–1999) (Figs 1 and S2). In its earliest stages, it was discovered in Japan, South Korea, and along the eastern coast of China (Liaoning, Shanghai, and Zhejiang); it then spread to Taiwan and inland China. Most GIb strains identified within the past 5 years (2016–2021), especially for strains of Northern China, belong to clade 2, particularly in the emerging sub-clade, with a TMRCA of 2005 (95% HPD: 2003–2007; S2 Fig). Exponential growth of the emerging clade is shown in Fig 2D.

### Spatial expansion of JEV GI in Asia

**Fig 3:**
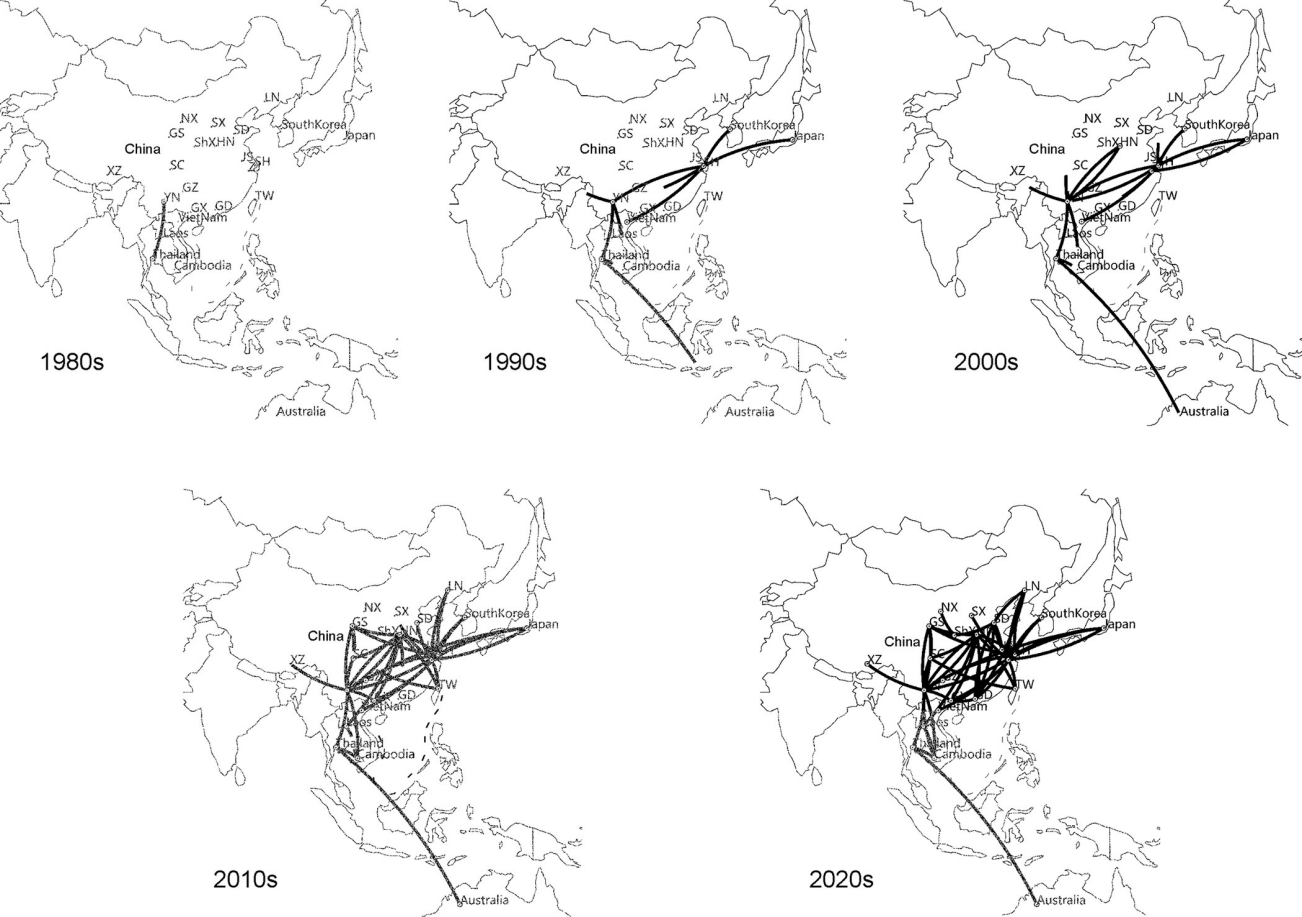
Spatial dynamics of JEV GI spread over the past five decades. Circles indicate sampling locations. Lines between locations represent branches of the maximum clade credibility (MCC) tree along which the relevant location transition occurred. The base layer of the modified maps are sourced from Natural Earth, and download in GeoJSON format from website (https://geojson-maps.ash.ms/).

Next, we investigated the spread of JEV GI in Asia. Our analyses identified Thailand as the location of the JEV GI common ancestor; they indicated a dynamic pattern of JEV GI movement in Asia, initially dominated by viral dispersal from Southeast Asia toward other population centers (Fig 3). By 1980, JEV GI strains were transmitted only within Southeast Asia and Yunnan Province in China. As shown in Fig 1, GIa strains were circulating at this stage. During the 1990s, JEV GI experienced exponential growth, GIb has replaced GIa as the dominate causative strain (Fig 2A); long-distance movement occurred to the north and south (Fig 3). JEV GI circulated in many regions of East Asia including Japan, South Korea, and Shanghai Province in China. By 2000, JEV GI had begun to spread deep into inland China, particularly in southern China; most Chinese provinces had experienced this JEV GI epidemic by 2010. In 2020, JEV GI was more active in northern China than in southern China (Figs 1 and 3).

### Selection analyses

We screened for sites under selection using different models implemented in the HYPHY framework. Evidence for both pervasive and episodic selection was found in different genes using different methods. Individual sites under pervasive selection were identified in the NS1, NS2a and NS5 proteins. Codons 75 and 215 of the NS1 protein, codons 14 and 364 of the NS5 protein were evolving under positive selection according to the BUSTED model; Codons 151 of the NS2a protein was evolving under positive selection according to the FEL model; Codon 175 of the NS1, coden 151 of the NS2a, codon 250 of the NS4b and codon 277 of the NS5 protein were evolving under positive selection according to the FUBAR model.

MEME analysis of sites under selection identified codon 120 in the E protein, codons 75 and 215 of the NS1 protein, codons 151 of NS2a protein, codon 48 and 250 of NS4b, codons 14, 277 and 364 of the NS5 protein (Table 1). Among these sites, seven of ten were identified using two or more methods.

**Table 1:** Evidence for adaptive selection on the full open reading frame (ORF) of the JEV genome according to different assessment models.

Gene	Length	Pervasive selection								Episodic selection			
		Gene-wise(BUSTED)		Site-wise(SLAC)		Site-wise(FEL)		Site-wise(FUBAR)		Site-wise(MEME)		Branch-site model(aBSREL)	
		P<0.05	Sites	P<0.05	Sites	P<0.05	Sites	P<0.05	Sites	P<0.05	Sites	P<0.05	Sites
C	127	No	-	No	-	No	-	No	-	No	-	No	-
PrM	167	No	-	No	-	No	-	No	-	No	-	No	-
E	500	No	-	No	-	No	-	No	-	Yes	120	Yes	-
NS1	325	Yes	75,215	No	-	No	-	Yes	175	Yes	75,215	No	-
NS2a	227	No	-	No	-	Yes	151	Yes	151	Yes	151	No	-
NS2b	131	No	-	No	-	No	-	No	-	No	-	No	-
NS3	619	No	-	No	-	No	-	No	-	No	-	No	-
NS4a	126	No	-	No	-	No	-	No	-	No	-	No	-
NS4b	255	No	-	No	-	No	-	Yes	250	Yes	48,250	No	-
NS5	905	Yes	14,364	No	-	No	-	Yes	277	Yes	14,277,364	No	-

aBSREL, adaptive Branch-Site Random Effects Likelihood; BUSTED, Branch site Unrestricted Statistical Test for Episodic Diversification; FEL, Fixed-Effects Likelihood; FUBAR, Fast Unconstrained Bayesian Approximation; MEME, Mixed-Effects Likelihood; SLAC, Single-Likelihood Ancestor Counting.

### Amino acid markers and corresponding nucleotide changes between different clades of GI

Comparison of GI genomes revealed that multiple amino acid sites show polymorphisms among evolutionary clades. Eight amino acid markers were found between GIa and GIb. Amino acid polymorphism sites prM-75F, E-129T, E-141V, NS2a-105M, NS2a-151T, NS3-345E, NS5-438D, and NS5-878V were identified as GIa virus markers; amino acid polymorphism sites prM-75Y, E-129M, E-141I, NS2a-105V, NS2a-151A/V, NS3-345E, NS5-438N, and NS5-878I were identified as GIb virus markers; amino acid polymorphism sites NS3-237K, NS4b-24S, and NS5287K were identified as GIb clade 2 virus markers; and amino acid polymorphism sites NS2a-151V and NS4b-20K were identified as markers of an emerging sub-clade; all corresponding conserved nucleotide changes were list in Table 2.

**Table 2:** Amino acid markers and corresponding nucleotide changes identified for different clades.of JEV GI.

Subtype	Clade	Subclade	prM	E	NS1	NS2a	NS3	NS4b	NS5
GIa			75F(TTC)	129T(ACA), 141V(GTA)	175D(GAC)	105M(ATG), 151T(ACC)	123I(ATT), 237R(AGA), 354E(GAA)	20R(AGG), 24P(CCA)	287R(AGA), 438D(GAT), 878V(GTC)
GIb	GIb clade 1		75Y(TAC)	129M(ATG), 141I(ATA)	175N(AAC)	105V(GTG), 151A(GCC)	123V(GTT), 237R(AGA), 354D(GAC)	20R(AGG), 24P(CCA)	287R(AGA), 438N(AAT), 878I(ATC)
	GIb clade 2		75Y(TAC)	129M(ATG), 141I(ATA)	175N(AAC)	105V(GTG),151A(GCC)	123I(ATT), 237K(AAA),354D(GAC)	20R(AGG), 24S(TCA)	287K(AAA), 438N(AAT),878I(ATC)
		Clade 2, emerging variant	75Y(TAC)	129M(ATG), 141I(ATA)	175D(GAC)	105V(GTG),151V(GTC)	123I(ATT), 237K(AAA),354D(GAC)	20K(AAG), 24S(TCA)	287K(AAA), 438N(AAT),878I(ATC)

## Discussion

JEV GI virus strains were first discovered in the 1990s; they replaced JEV GIII as the dominant genotype in Asia after 2000 [10–15]. Although previous studies have explored the spatial and evolutionary dynamics of current JEV transmission in Asia [6, 7, 12, 25–28], a shortage of full-length genomic data, particularly recently sampled JEV GI sequences, has limited our ability to fully examine the new epidemic trends and evolutionary dynamics of JEV GI transmission in the past 10years. One study used 234 JEV E gene sequences to analyze the division of GIa and GIb; GIa remained confined to tropical Asia, GIb displace GIII as the dominant genotype throughout Asia [25]. A concurrent study of 678 JEV E gene sequences observed no divergence of GIa and GIb [26]. The TMRCA values calculated for JEV GI also differed between studies [6, 7, 12, 25–28].

In this study, we generated and analyzed 18 new near full length JEV genomic sequences from mosquitoes that had been collected in several Chinese provinces from 2016 to 2019, greatly expanding the observed viral genetic diversity of recent year. Another 113 published sequences from GenBank were included in the final dataset. The generated genomic data provided a more detailed understanding of the progression of GIb in Asia during the past decades; we also revealed the timing, source, and likely routes of JEV transmission and dispersion throughout China during that period. Determining the large-scale spread of JEV GI and its geographic hot spots is essential for predicting and preventing potential spillover events.

Phylogeography based on 131 full ORFs of JEV GIb sequences allowed us to clearly determine the division between GIa and GIb [25]. From the mid-20th century to 1980, GIa has remained geographically confined to a limited region. GIa is mainly found in the tropics and subtropics (Thailand, Cambodia, Australia, and Yunnan Province), it is still circulating but has caused no mass epidemics since the origin of GI. A previous study found that GI strains in Yunnan Province are mainly of the GIb subtype [41]; in the present study, we found evidence that GIa remains prevalent in this area. These results indicate the co-prevalence of GIa and GIb within the region. In contrast, GIb showed very strong transmission capability; it originated in the subtropics and then replaced GIII as the dominant JEV genotype throughout Asia in the 1990s, where it spread widely for more than 20 years and caused serious public health concerns. Our investigation of the evolution dynamics of JEV GIb showed a gradual shift in the epidemic strains of JEV GI circulating in Asia. Viral strains show distinct molecular characteristics according to time period and location. GIb circulated within East Asia during the past 30 years and some strains clustered together (particularly early GIb strains that have gradually become less motile); the two most active GIb clades that trigger epidemics showed spatiotemporal differences in their spread. During the past decade, clade 1 strains have shown greater transmission activity in southern China, while clade 2 strains have been more active in northern China. The substitution rate of the GI full ORF was estimated to be 5.94 × 10^−4^ s/s/y (95% BCI: 5.03–6.94 × 10^−4^ s/s/y). We identified amino acid markers for different subclades of JEV GI, including eight amino acid markers between GIa and GIb and two for the most active emerging clade (Table 2). Among these sites, two amino acid markers of different clades (NS1-175, NS2a-151) were found to be under positive selection, but the functional impact of these mutations on viral proteins and their phenotypic significance remains undocumented. Notably, NS2a (T151A) mutation has also been observed in circulating Zika virus strains [42]. The evolutionary genomics of JEV GI in Asia during past 30 years are presumably driven by a complex combination of factors such as host–vector adaptability, vector competence, and virus replicative ability. Thus far, there have been few evolutionary and phenotypic studies of different subclades of JEV GI. The larger genome information and dataset developed in this study contributed to the identification of subclades in our phylogenetic analysis; our method allowed detailed examination of the transmission trends of different subclades. These epidemic trends indicated differences in virus adaptability under natural selection.

Recent studies have observed the change of epidemiological and clinical characteristics of JE in Northern China. There have been reported several adult JE epidemics occurred in this area after 2000 [21, 23, 24, 43, 44]. Causal link between Guillain–Barré syndrome and JEV infection have also been reported [22, 45]. This study focused on describing the molecular characteristics of JEV strains in the areas where the epidemic trend and clinical characteristics have recently changed. Our team previously isolated GIb strain NX1889 from a patient during an outbreak of JE in 2018 [21] and the new isolated strains LK1808, GS1943, SX19117, SX31-24 et al identified in the present study from Northern China all belonged to the emerging clade (NS2a-151V, NS4b-20K). These findings underscore the importance of continued surveillance for JE causative strain variation as well as their correlation with epidemic characteristics of JE.

When supported by the analysis of samples collected at different time points and/or locations, phylodynamics can be used to describe trends in epidemic spread [46–48]. Although this phylogeographic study obtained valuable information, these data should be interpreted in light of its limitations. Because we lack data regarding pre-1970 isolates of JEV strains, we cannot exclude the possibility of bias in our GI epidemic origin estimates. We included all available GIa sequences from GenBank to the extent possible, but the number of sequences was limited. Bayesian skyline analysis revealed that JEV GIa groups exhibited no significant growth during the past 40 years. The wider availability of JEV E genes has led to their use in multiple phylodynamics studies [12, 25, 26]; however, E genes cannot be used to assess whether diversification and/or directional selection in other regions of the genome may have facilitated viral genome adaptation to its environment. Because of the greater availability of JEV ORF sequence data in this study, we utilized a dataset that contained sequence information derived from the full ORF.

Overall, this study provides valuable insights into the evolution, spatiotemporal spread, and amino acid markers of JEV GI, highlights the importance of continued surveillance of JEV strains in humans and mosquitoes in nature, in both epidemic and non-epidemic regions of China, to quantify the risks of new outbreaks within the region. Our results indicate that genomic data can be employed to assist public health services in monitoring and understanding the diversity of circulating mosquito-borne viruses.

## Supporting information

S1 Fig Root-to-tip regression analyses of phylogenetic temporal signals. (TIF)

S2 Fig Mean and 95% Bayesian credible intervals (BCIs) of the time of the most recent common ancestor (TMRCA) of each group. (TIF)

S3 Fig Visualization of full open reading frames of Japanese encephalitis virus (JEV) genotype I (GI) variants. The reference is JS-1 strain (GenBank accession number: KX357114) isolated from Mosquito in 2015.
(TIF)

S1 Table Sample information for Japanese encephalitis virus complete genome sequences identified in the study. (DOCX)

S2 Table Sample information for Japanese encephalitis virus complete genome sequences enrolled in the study. (DOCX)

S3 Table Location phylogeographic analysis of JEV genotype I (GI). (DOCX)
