# Supplementary figures and images for: Tracing the spatiotemporal phylodynamics of Japanese encephalitis virus genotype I throughout Asia and the western Pacific
Source: PLoS Negl Trop Dis. 2023 Apr 13;17(4):e0011192. doi: 10.1371/journal.pntd.0011192 (PMC10128984; doi:10.1371/journal.pntd.0011192)

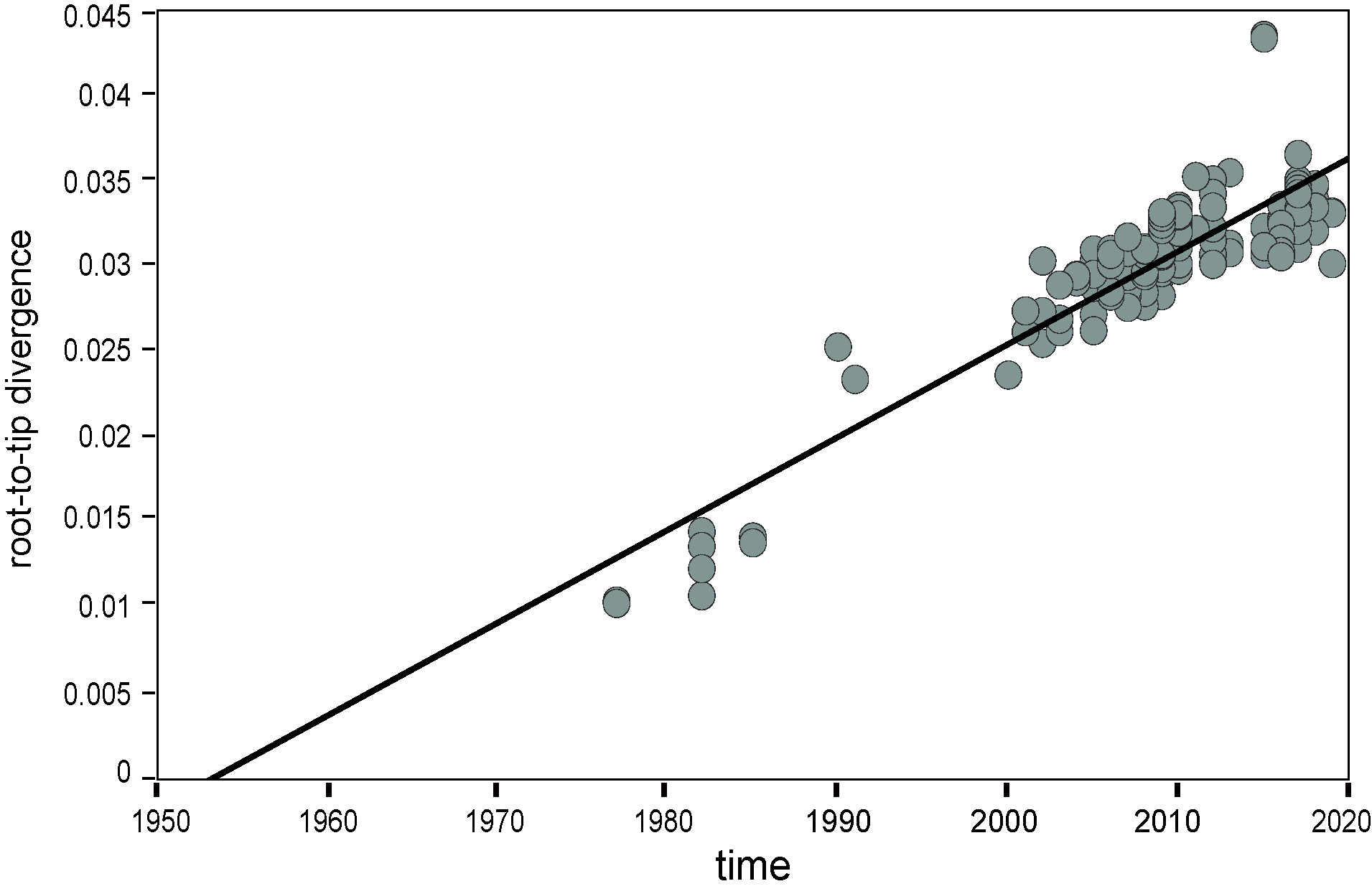

Supplement: S1 Fig — (TIF) [file pntd.0011192.s001.tif]

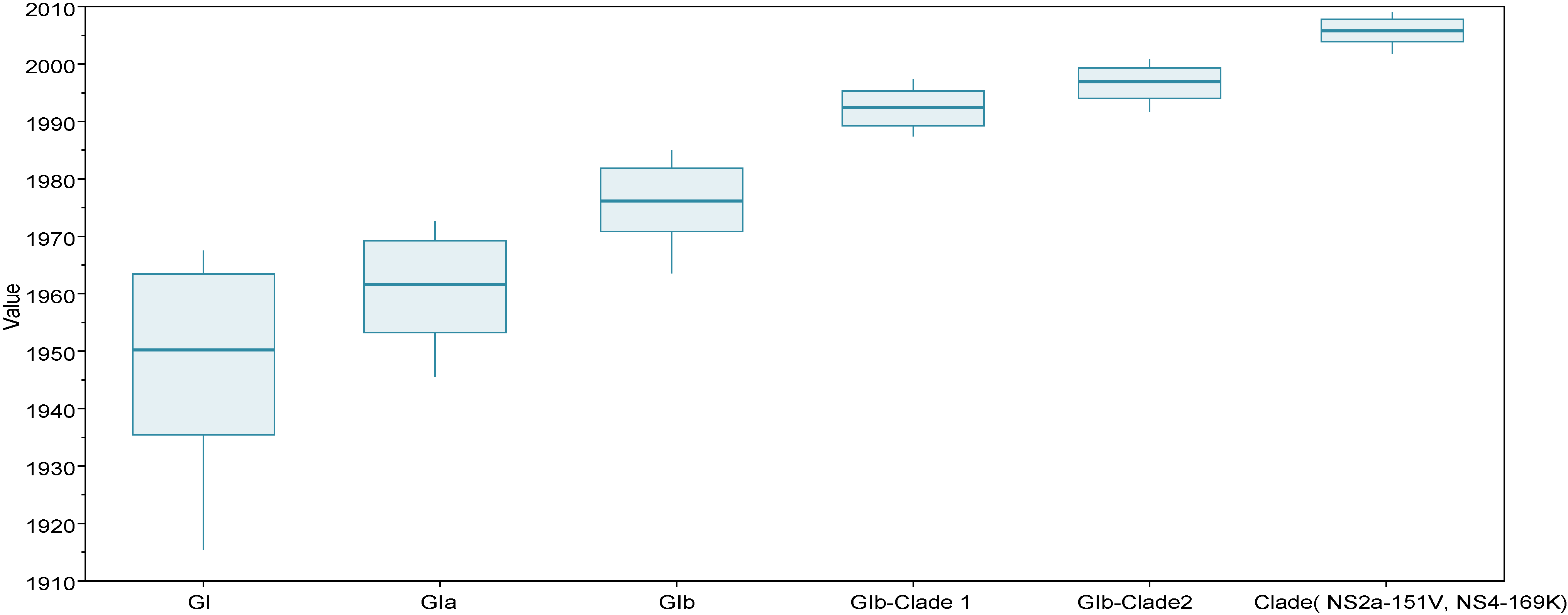

Supplement: S2 Fig — (TIF) [file pntd.0011192.s002.tif]

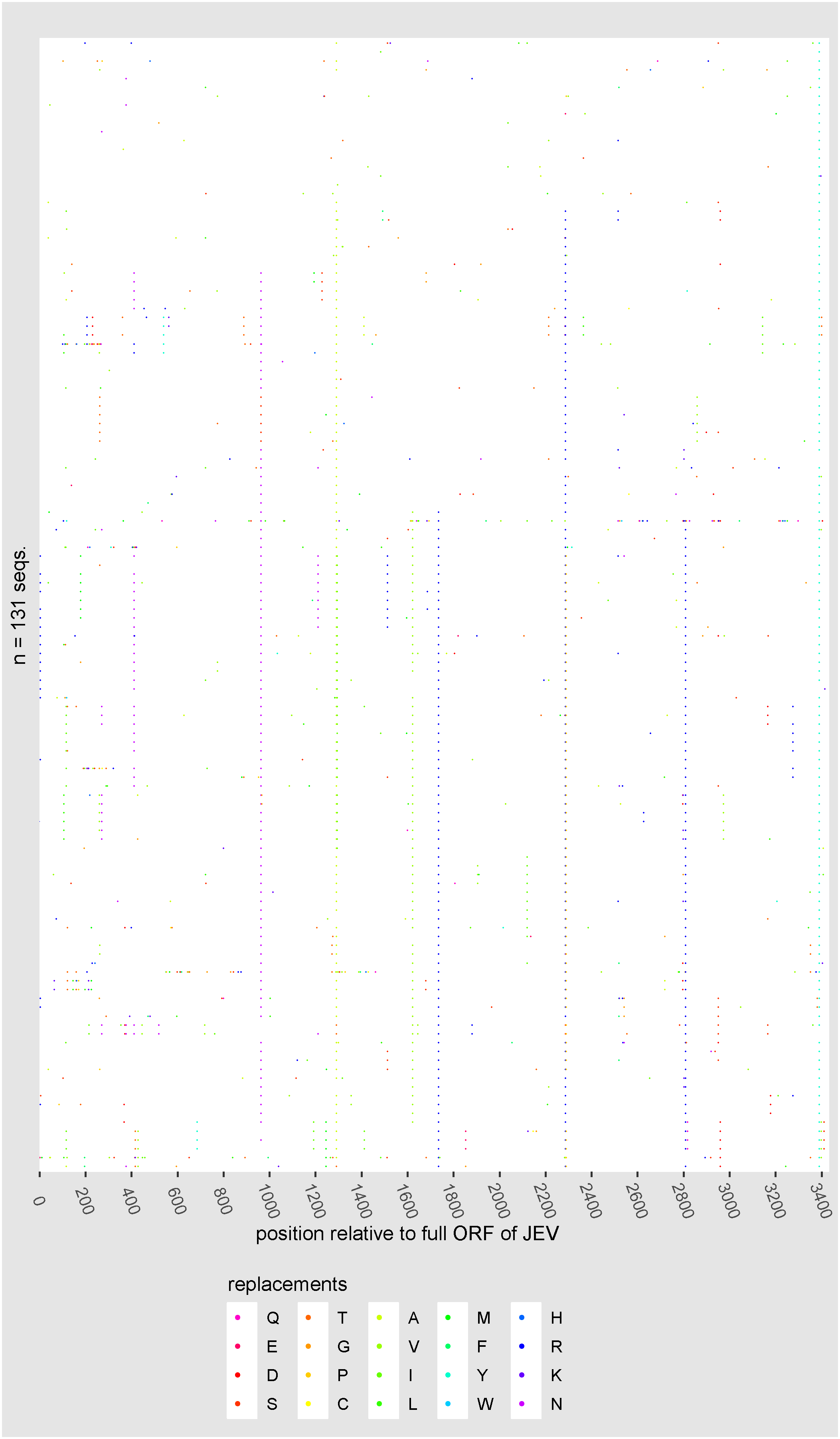

Supplement: S3 Fig — The reference is JS-1 strain (GenBank accession number: KX357114) isolated from Mosquito in 2015. (TIF) [file pntd.0011192.s003.tif]
