# Supplementary material for: Tracing the spatiotemporal phylodynamics of Japanese encephalitis virus genotype I throughout Asia and the western Pacific
Source: PLoS Negl Trop Dis. 2023 Apr 13;17(4):e0011192. doi: 10.1371/journal.pntd.0011192 (PMC10128984; doi:10.1371/journal.pntd.0011192)
Supplement: S1 Table — (DOCX) [file pntd.0011192.s004.docx]

Table S1. Sample information for Japanese encephalitis virus complete genome sequences identified in the study

| Strain | Year | Country | Province (Abbreviation) | Host | Sample Type | Sequencing approach | Accession number |
| --- | --- | --- | --- | --- | --- | --- | --- |
| SDWS1607 | 2016 | China | Shandong (SD) | Culex tritaeniorhynchus | Viral isolate | Sanger sequencing | OM572535 |
| SDJN1633 | 2016 | China | Shandong (SD) | Culex tritaeniorhynchus | Viral isolate | Sanger sequencing | OM572533 |
| SDJN1646 | 2016 | China | Shandong (SD) | Culex tritaeniorhynchus | Viral isolate | Sanger sequencing | OM572534 |
| SDWS1619 | 2016 | China | Shandong (SD) | Culex tritaeniorhynchus | Viral isolate | Sanger sequencing | OM572536 |
| XYM17305 | 2017 | China | Yunnan (YN) | Culex tritaeniorhynchus | Viral isolate | NGS | OM572542 |
| XYM17198 | 2017 | China | Yunnan (YN) | Culex tritaeniorhynchus | Viral isolate | NGS | OM572544 |
| XYM17227 | 2017 | China | Yunnan (YN) | Culex tritaeniorhynchus | Viral isolate | NGS | OM572543 |
| GX-059sd | 2017 | China | Guangxi (GX) | Culex tritaeniorhynchus | Viral isolate | Sanger sequencing | OM572546 |
| LN1814 | 2018 | China | Liaoning (LN) | Culex tritaeniorhynchus | Viral isolate | NGS | OM572545 |
| LN1821 | 2018 | China | Liaoning (LN) | Culex tritaeniorhynchus | Viral isolate | NGS | OM572547 |
| LN1861 | 2018 | China | Liaoning (LN) | Culex tritaeniorhynchus | Viral isolate | NGS | OM572548 |
| LK1808 | 2018 | China | Shandong (SD) | Mosquito | Mosquitoes homogenized supernatant | NGS and Sanger sequencing | OM572537 |
| SX31-24 | 2018 | China | Shaanxi (ShX) | Mosquito | Mosquitoes homogenized supernatant | NGS and Sanger sequencing | OM572541 |
| ZJ1844 | 2018 | China | Zhejiang (ZJ) | Culex tritaeniorhynchus | Viral isolate | NGS | OM572549 |
| ZJ1866 | 2018 | China | Zhejiang (ZJ) | Culex tritaeniorhynchus | Viral isolate | NGS | OM572550 |
| JN19-1 | 2019 | China | Shandong (SD) | Mosquito | Mosquitoes homogenized supernatant | NGS and Sanger sequencing | OM572538 |
| GS1943 | 2019 | China | Gansu (GS) | Culex tritaeniorhynchus | Mosquitoes homogenized supernatant | NGS and Sanger sequencing | OM572539 |
| SX19117 | 2019 | China | Shaanxi (ShX) | Culex tritaeniorhynchus | Mosquitoes homogenized supernatant | NGS and Sanger sequencing | OM572540 |
