# Supplementary material for: Tracing the spatiotemporal phylodynamics of Japanese encephalitis virus genotype I throughout Asia and the western Pacific
Source: PLoS Negl Trop Dis. 2023 Apr 13;17(4):e0011192. doi: 10.1371/journal.pntd.0011192 (PMC10128984; doi:10.1371/journal.pntd.0011192)
Supplement: S2 Table — (DOCX) [file pntd.0011192.s005.docx]

Table S2. Sample information for Japanese encephalitis virus complete genome sequences enrolled in the study

| No. | Strain | Year | Country | Province (Abbreviation) | Host | Accession number |
| --- | --- | --- | --- | --- | --- | --- |
| 1 | JS_1 | 2015 | China | Jiangsu (JS) | Mosquito | KX357114 |
| 2 | anheal | 2017 | China | Shandong (SD) | Seal | MH165313 |
| 3 | NX1889 | 2018 | China | Ningxia (NX) | Human | MT134112 |
| 4 | 131V | 2007 | China | Guangxi (GX) | Human | GU205163 |
| 5 | 1991 | 1991 | South Korea |  | Mosquito | KU705224 |
| 6 | 90VN70 | 1990 | Vietnam |  | Human | HM228921 |
| 7 | BL06_50 | 2006 | China | Guangxi (GX) | Mosquito | JF706270 |
| 8 | BL06_54 | 2006 | China | Guangxi (GX) | Mosquito | JF706271 |
| 9 | DH10M585 | 2010 | China | Yunnan (YN) | Mosquito | KT957421 |
| 10 | DH10M865 | 2010 | China | Yunnan (YN) | Mosquito | KT229572 |
| 11 | DH10M978 | 2010 | China | Yunnan (YN) | Mosquito | KT229573 |
| 12 | DHL10M62 | 2010 | China | Yunnan (YN) | Mosquito | KT229575 |
| 13 | GSBY0801 | 2008 | China | GanSu (GS) | Mosquito | JF706274 |
| 14 | GSBY0804 | 2008 | China | GanSu (GS) | Mosquito | JN381844 |
| 15 | GSBY0810 | 2008 | China | GanSu (GS) | Mosquito | JN381840 |
| 16 | GSBY0816 | 2008 | China | GanSu (GS) | Mosquito | JN381842 |
| 17 | GSBY0827 | 2008 | China | GanSu (GS) | Mosquito | JN381845 |
| 18 | GSBY0861 | 2008 | China | GanSu (GS) | Mosquito | JN381833 |
| 19 | GZ56 | 2008 | China | Guizhou (GZ) | Human | HM366552 |
| 20 | HEN0701 | 2007 | China | Henan (HN) | Swine | FJ495189 |
| 21 | HL2010_2 | 2010 | China | Taiwan | Mosquito | JQ031753 |
| 22 | HN06129 | 2006 | China | Henan (HN) | Mosquito | JF706277 |
| 23 | HN0621 | 2006 | China | Henan (HN) | Mosquito | JN381830 |
| 24 | HN0626 | 2006 | China | Henan (HN) | Mosquito | JN381837 |
| 25 | Bo/Aichi/1/2010 | 2010 | Japan |  | Cattle | AB853904 |
| 26 | Bo/Miyazaki/1/2009 | 2009 | Japan |  | Cattle | AB830335 |
| 27 | CNS769/Laos/2009 | 2009 | Laos |  | Human | KC196115 |
| 28 | eq/Tottori/2003 | 2003 | Japan |  | Horse | AB594829 |
| 29 | MQ/Yamaguchi/2013_1 | 2013 | Japan |  | Mosquito | AB981183 |
| 30 | MQ/Yamaguchi/2013_2 | 2013 | Japan |  | Mosquito | AB981184 |
| 31 | MQ/Yamaguchi/804/2016 | 2016 | Japan |  | Mosquito | LC461957 |
| 32 | sw/Mie/40/2004 | 2004 | Japan |  | swine | AB241118 |
| 33 | sw/Mie/41/2002 | 2002 | Japan |  | swine | AB241119 |
| 34 | sw/Okinawa/127/2012 | 2012 | Japan |  | Swine | AB920399 |
| 35 | Taiwan/H10100739/H/2012 | 2012 | China | Taiwan | Human | KF667324 |
| 36 | Taiwan/TC0906a/M/2009 | 2009 | China | Taiwan | Mosquito | KF667319 |
| 37 | Taiwan/TC0906d/M/2009 | 2009 | China | Taiwan | Mosquito | KF667320 |
| 38 | Taiwan/TC1006h/M/2010 | 2010 | China | Taiwan | Mosquito | KF667321 |
| 39 | Taiwan/TC1106i/M/2011 | 2011 | China | Taiwan | Mosquito | KF667322 |
| 40 | Taiwan/TN1205a/M/2012_2 | 2012 | China | Taiwan | Mosquito | KF667325 |
| 41 | Taiwan/TN1205b/M/2012 | 2012 | China | Taiwan | Mosquito | KF667326 |
| 42 | Taiwan/TPC0806c/M/2008 | 2008 | China | Taiwan | Mosquito | KF667316 |
| 43 | Taiwan/TPC0906ah/M/2009 | 2009 | China | Taiwan | Mosquito | KF667318 |
| 44 | Taiwan/YL0806f/M/2008 | 2008 | China | Taiwan | Mosquito | KF667317 |
| 45 | Taiwan/YL1106b/M/2011_2 | 2011 | China | Taiwan | Mosquito | KF667327 |
| 46 | Taiwan/YL1206a/M/2012_2 | 2012 | China | Taiwan | Mosquito | KF667323 |
| 47 | JX61 | 2008 | China | Zhejiang (ZJ) | Swine | GU556217 |
| 48 | K05GS | 2005 | South Korea |  | Mosquito | KR908702 |
| 49 | LN02_102 | 2002 | China | Liaoning (LN) | Mosquito | JF706278 |
| 50 | LN0716 | 2007 | China | Liaoning (LN) | Mosquito | JN381849 |
| 51 | ME802 | 2013 | Cambodia |  | Human | KY927819 |
| 52 | SH17M-07 | 2007 | China | Shanghai (SH) | Mosquito | EU429297 |
| 53 | SC0412 | 2004 | China | Sichuan (SC) | Mosquito | JN381839 |
| 54 | SC0415 | 2004 | China | Sichuan (SC) | Mosquito | JN381838 |
| 55 | SCCZ | 2010 | China | Sichuan (SC) | Mosquito | KU351667 |
| 56 | SCYA2012 | 2012 | China | Sichuan (SC) | Swine | KU508408 |
| 57 | SCYA2012 | 2012 | China | Sichuan (SC) | Swine | KU508409 |
| 58 | SCYA201201 | 2012 | China | Sichuan (SC) | Swine | KM658163 |
| 59 | SD0810 | 2009 | China | Shandong (SD) | Mosquito | JF706286 |
| 60 | SD12 | 2015 | China | Shanghai (SH) | Swine | MH753127 |
| 61 | SH03103 | 2003 | China | Shanghai (SH) | Mosquito | JN381847 |
| 62 | SH03105 | 2003 | China | Shanghai (SH) | Mosquito | JN381846 |
| 63 | SH2 | 2016 | China | Shanghai (SH) | Mosquito | MH753133 |
| 64 | SH53 | 2001 | China | Shanghai (SH) | Mosquito | JN381850 |
| 65 | SH7 | 2016 | China | Shanghai (SH) | Mosquito | MH753129 |
| 66 | SH80 | 2001 | China | Shanghai (SH) | Mosquito | JN381848 |
| 67 | Sw/Mie/34/2004 | 2004 | Japan |  | Pig | AB698909 |
| 68 | Sw/Mie/51/2006 | 2006 | Japan |  | Pig | AB698905 |
| 69 | Sw/Mie/84/2005 | 2005 | Japan |  | Pig | AB698906 |
| 70 | Sw/Tokyo/373/2005 | 2005 | Japan |  | Pig | AB698907 |
| 71 | Sw/Tokyo/602/2005 | 2005 | Japan |  | Pig | AB698908 |
| 72 | SX09S_1 | 2009 | China | Shanxi (SX) | Swine | HQ893545 |
| 73 | TC2009_1 | 2009 | China | Taiwan | Mosquito | JF499790 |
| 74 | TC2009_3 | 2009 | China | Taiwan | Mosquito | JF499788 |
| 75 | MF124315 | 2016 | China | Shanghai (SH) | Swine | MF124315 |
| 76 | MF124316 | 2016 | China | Shanghai (SH) | Swine | MF124316 |
| 77 | MH184567 | 2017 | China | Guangdong (GD) | Mosquito | MH184567 |
| 78 | MH184568 | 2017 | China | Guangdong (GD) | Mosquito | MH184568 |
| 79 | MH184569 | 2017 | China | Guangdong (GD) | Mosquito | MH184569 |
| 80 | MH184570 | 2017 | China | Guangdong (GD) | Mosquito | MH184570 |
| 81 | MH184571 | 2017 | China | Guangdong (GD) | Mosquito | MH184571 |
| 82 | MH184572 | 2017 | China | Guangdong (GD) | Mosquito | MH184572 |
| 83 | MH184573 | 2017 | China | Guangdong (GD) | Midge | MH184573 |
| 84 | MH184574 | 2017 | China | Guangdong (GD) | Mosquito | MH184574 |
| 85 | MH184575 | 2017 | China | Guangdong (GD) | Mosquito | MH184575 |
| 86 | MH184576 | 2017 | China | Guangdong (GD) | Mosquito | MH184576 |
| 87 | XJ69 | 2007 | China | Zhejiang (ZJ) | Mosquito | EU880214 |
| 88 | XJP613 | 2007 | China | Zhejiang (ZJ) | Mosquito | EU693899 |
| 89 | XZ0938 | 2009 | China | Tibet (XZ) | Mosquito | HQ652538 |
| 90 | YL2009_4 | 2009 | China | Taiwan | Mosquito | JF499789 |
| 91 | YN05124 | 2005 | China | Yunnan (YN) | Mosquito | JF706281 |
| 92 | YN05155 | 2005 | China | Yunnan (YN) | Mosquito | JN381852 |
| 93 | YN0623 | 2006 | China | Yunnan (YN) | Mosquito | JN381836 |
| 94 | YN0911 | 2009 | China | Yunnan (YN) | Mosquito | JF706267 |
| 95 | YN0967 | 2009 | China | Yunnan (YN) | Mosquito | JF706268 |
| 96 | YN09M57 | 2009 | China | Yunnan (YN) | Mosquito | KT229574 |
| 97 | YNTC07018 | 2007 | China | Yunnan (YN) | Mosquito | KT957420 |
| 98 | YNTC07172 | 2007 | China | Yunnan (YN) | Mosquito | KT957419 |
| 99 | ZJ10_10 | 2010 | China | Zhejiang (ZJ) | Mosquito | KY650727 |
| 100 | ZJ10_7 | 2010 | China | Zhejiang (ZJ) | Mosquito | KY650726 |
| 101 | C14_B3 | 2015 | Cambodia |  | Swine | KY927817 |
| 102 | D03_B9 | 2015 | Cambodia |  | Swine | KY927818 |
| 103 | B_0860/82 | 1982 | Thailand |  | Swine | GQ902058 |
| 104 | BN82215 | 1982 | China | Yunnan (YN) | Mosquito | KT957423 |
| 105 | M28 | 1977 | China | Yunnan (YN) | Mosquito | JF706279 |
| 106 | M28 | 1977 | China | Yunnan (YN) | Mosquito | KT957422 |
| 107 | 1070/82 (Subin) | 1982 | Thailand |  | Human | GQ902059 |
| 108 | 4790-85 | 1982 | Thailand |  | Mosquito | GQ902060 |
| 109 | B-1381-85 | 1985 | Thailand |  | Swine | GQ902061 |
| 110 | 4790-85 | 1985 | Thailand |  | Human | GQ902062 |
| 111 | TS00 | 2000 | Australia |  | Swine | MT253732 |
| 112 | Badu2002 | 2002 | Australia |  | Swine | MT253733 |
| 113 | C081 | 2015 | Cambodia |  | Human | KY927816 |
