# Supplementary material for: Tracing the spatiotemporal phylodynamics of Japanese encephalitis virus genotype I throughout Asia and the western Pacific
Source: PLoS Negl Trop Dis. 2023 Apr 13;17(4):e0011192. doi: 10.1371/journal.pntd.0011192 (PMC10128984; doi:10.1371/journal.pntd.0011192)
Supplement: S3 Table — (DOCX) [file pntd.0011192.s006.docx]

Table S3. Location phylogeographic analysis of JEV GI.

| Group | Location | Posterior Probability |
| --- | --- | --- |
| JEV GI | Thailand | 0.4074 |
|  | Vietnam | 0.0112 |
|  | South Korea | 0.0118 |
|  | Australia | 0.0085 |
|  | Laos | 0.0066 |
|  | Japan | 0.0191 |
|  | Cambodia | 0.0335 |
|  | China（Liaoning) | 0.0072 |
|  | China (Tibet) | 0.0204 |
|  | China (Shaanxi) | 0.0046 |
|  | China (Shanxi) | 0.0033 |
|  | China (Henan) | 0.0210 |
|  | China (Jiangsu) | 0.0085 |
|  | China (Ningxia) | 0.0059 |
|  | China (Gansu) | 0.0223 |
|  | China (Sichuan) | 0.0105 |
|  | China (Zhejiang) | 0.0105 |
|  | China (Guangxi) | 0.0164 |
|  | China (Shandong) | 0.0079 |
|  | China (Guizhou) | 0.0066 |
|  | China (Yunnan) | 0.2693 |
|  | China (Shanghai) | 0.0723 |
|  | China (Guangdong) | 0.0079 |
|  | China (Taiwan) | 0.0072 |
